# A Technological Innovation to Reduce Prescribing Errors Based on Implementation Intentions: The Acceptability and Feasibility of MyPrescribe

**DOI:** 10.2196/humanfactors.7153

**Published:** 2017-08-01

**Authors:** Chris Keyworth, Jo Hart, Hong Thoong, Jane Ferguson, Mary Tully

**Affiliations:** ^1^ Manchester Centre for Health Psychology Division of Psychology and Mental Health, School of Health Sciences, Faculty of Biology, Medicine and Health University of Manchester, Manchester Academic Health Science Centre Manchester United Kingdom; ^2^ Division of Medical Education School of Medical Sciences, Faculty of Biology, Medicine and Health University of Manchester, Manchester Academic Health Science Centre Manchester United Kingdom; ^3^ Great Ormond Street Hospital for Children NHS Foundation Trust London United Kingdom; ^4^ Health Management Group Alliance Manchester Business School, Faculty of Humanities University of Manchester, Manchester Academic Health Science Centre Manchester United Kingdom; ^5^ Division of Pharmacy and Optometry School of Health Sciences, Faculty of Biology, Medicine and Health University of Manchester, Manchester Academic Health Science Centre Manchester United Kingdom

**Keywords:** drug prescribing, behavior and behavior mechanisms, clinical competence, qualitative research, mobile applications, pharmacists, patient safety, telemedicine

## Abstract

**Background:**

Although prescribing of medication in hospitals is rarely an error-free process, prescribers receive little feedback on their mistakes and ways to change future practices. Audit and feedback interventions may be an effective approach to modifying the clinical practice of health professionals, but these may pose logistical challenges when used in hospitals. Moreover, such interventions are often labor intensive. Consequently, there is a need to develop effective and innovative interventions to overcome these challenges and to improve the delivery of feedback on prescribing. Implementation intentions, which have been shown to be effective in changing behavior, link critical situations with an appropriate response; however, these have rarely been used in the context of improving prescribing practices.

**Objective:**

Semistructured qualitative interviews were conducted to evaluate the acceptability and feasibility of providing feedback on prescribing errors via MyPrescribe, a mobile-compatible website informed by implementation intentions.

**Methods:**

Data relating to 200 prescribing errors made by 52 junior doctors were collected by 11 hospital pharmacists. These errors were populated into MyPrescribe, where prescribers were able to construct their own personalized action plans. Qualitative interviews with a subsample of 15 junior doctors were used to explore issues regarding feasibility and acceptability of MyPrescribe and their experiences of using implementation intentions to construct prescribing action plans. Framework analysis was used to identify prominent themes, with findings mapped to the behavioral components of the COM-B model (capability, opportunity, motivation, and behavior) to inform the development of future interventions.

**Results:**

MyPrescribe was perceived to be effective in providing opportunities for critical reflection on prescribing errors and to complement existing training (such as junior doctors’ e-portfolio). The participants were able to provide examples of how they would use “If-Then” plans for patient management. Technology, as opposed to other methods of learning (eg, traditional “paper based” learning), was seen as a positive advancement for continued learning.

**Conclusions:**

MyPrescribe was perceived as an acceptable and feasible learning tool for changing prescribing practices, with participants suggesting that it would make an important addition to medical prescribers’ training in reflective practice. MyPrescribe is a novel theory-based technological innovation that provides the platform for doctors to create personalized implementation intentions. Applying the COM-B model allows for a more detailed understanding of the perceived mechanisms behind prescribing practices and the ways in which interventions aimed at changing professional practice can be implemented.

## Introduction

Despite being one of the most common interventions that patients receive when admitted to a hospital, prescribing is rarely an error-free process [1,2]. Prescribing errors place a substantial burden on the health system and can result in preventable adverse drug events, prolonged hospital stay, and an increased risk of death. The cost to the National Health Service (NHS) in England is in excess of £750 million annually [3].

The causes of prescribing errors are complex. Contributing factors include individual lack of knowledge and experience, lack of professional support focused on prescribing practices, and limitations in the work environment [2,4,5]. Consequently, there is a need to develop effective and innovative ways of improving prescribing practices. Foundation doctors are a particularly important professional group to target, as they order approximately 70% of hospital prescriptions and are twice as likely to make errors than the consultants [1].

Prescribers receive little feedback on their mistakes and ways to change future practice. In addition, the feedback that is provided is often irregular and insufficient [6]. A number of recent systematic reviews suggest that audit and feedback interventions may be an effective way of changing the behavior of health professionals [7-9] through improving performance and professional standards. A recent study examining the effectiveness of a pharmacist-led audit and feedback intervention found that it increased appropriate antimicrobial prescribing [10], suggesting that this may be an appropriate strategy for improving prescribing in general.

Once they receive feedback on their prescribing practices, the prescribers have to decide what to do differently in the future to change their behavior. Providing feedback alone has been shown to be less effective than feedback that includes both explicit targets and an action plan [7]. Implementation intentions or “If-Then” plans have been shown to be effective in changing behavior in general [11]. Our preliminary work has shown that workshops based on these psychological theories may be helpful in improving prescribing safety [10].

However, audit and feedback interventions on prescribing are rarely used in hospitals because of logistical difficulties such as problems identifying the prescriber from a signature alone [12]. In addition, it is important to identify ways in which we can deliver audit and feedback interventions in a busy clinical environment. Running workshops for patient-facing health professionals in hospitals is particularly difficult because of shift work [10]. More research is therefore needed to examine novel delivery methods specifically focused on applying audit and feedback to prescribing within hospitals. Technology-based interventions are particularly appealing as a delivery method, as they are perceived as helpful in numerous areas of clinical practice such as providing tailored information to patients [13], providing timely access to information to support practice [14,15], and emphasizing responsibility and competence relating to areas of clinical practice [15].

### Implementation Intentions

Theoretical approaches to behavior change in the context of prescribing behaviors creates an opportunity to develop interventions based on increasing awareness of mistakes and encouraging critical reflection [16]. Implementation intentions are “If-Then” plans that link a critical situation (“if”) with an appropriate response (“then”) [17]. They are a commonly used technique to address health behavior change and have been shown to have sustained effects on behavior change [18,19]. This method has been used successfully in a wide range of health contexts [18,20-22]. There have also been a number of successful applications of this approach in areas of health professional practice, including delivery of mental health services [23], improving clinical nursing practices [24], enhancing vaccination rates [25], as well as helping nurses and midwives incorporate healthy lifestyle behaviors in their own lives [26]. However, it remains unclear whether this approach can be used in the context of improving the prescribing practices of health professionals. An implementation-intentions−based intervention can be delivered via a technological platform without the need for debriefs with expert input. One of the aims of our study was to examine whether implementation intentions are perceived as an acceptable and feasible intervention delivery component for interventions aimed at improving prescribing practices.

### The COM-B System of Behavior

**Figure 1:**
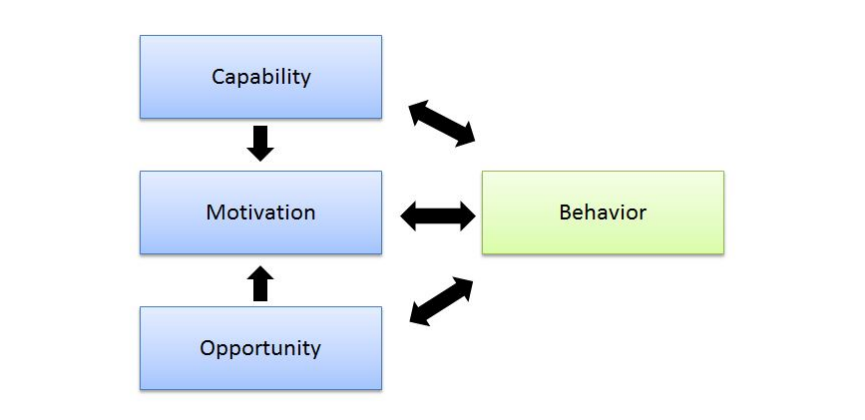
The COM-B (capability, opportunity, motivation, and behavior) model based on Michie et al.

The COM-B system [27] presented in Figure 1 [28] has been developed as a part of the behavior change wheel, designed to specifically inform intervention design [27,29]. The COM-B (capability, opportunity, motivation, and behavior) system proposes that engagement in behavior change occurs when one or more conditions are met. Individuals must have the capability to engage in the behavior, the opportunity to carry out a behavior, and the motivation to engage in the behavior rather than any other competing behaviors at the time. The model recognizes that behavior change is determined by an interacting system involving these different components [27]. The *capability* component includes both psychological and physical ability to carry out the behavior, *motivation* includes both reflective and automatic processes involved in initiation of the behavior, and *opportunity* includes the physical and social environment that facilitates the behavior change [27]. The COM-B model has been applied to health professional practice such as behavior change relating to test ordering behavior [30], identifying target behaviors associated with adult hearing aid fitting consultations [31], and examining the barriers and enablers to delivering health assessments [32] and writing discharge prescriptions [33].

Using the COM-B model allows theoretical insights to be used to formulate specific recommendations for intervention design [27]. The model also includes consideration of specific barriers and facilitators involved in the uptake of interventions and the subsequent behavior change. This study aims to examine the acceptability and feasibility of a novel technological innovation aimed at health professional behavior change, which is lowering the incidence of prescribing errors. As such, the COM-B model provides important insights into the barriers and facilitators to delivering interventions aimed at changing prescribing behavior as well as to inform the design of interventions.

### Aims

Through qualitative semistructured interviews with foundation doctors, this study addressed three specific aims: (1) to evaluate the acceptability and feasibility of providing prescribing error feedback via a technological innovation (MyPrescribe, a mobile-compatible website informed by implementation intentions), (2) to analyze and discuss the findings in the context of an established behavior change theory, the COM-B model, and (3) to outline a series of practical implications and recommendations for using MyPrescribe to change the prescribing behavior of health professionals involved in prescribing.

## Methods

### Development of MyPrescribe

**Figure 2:**
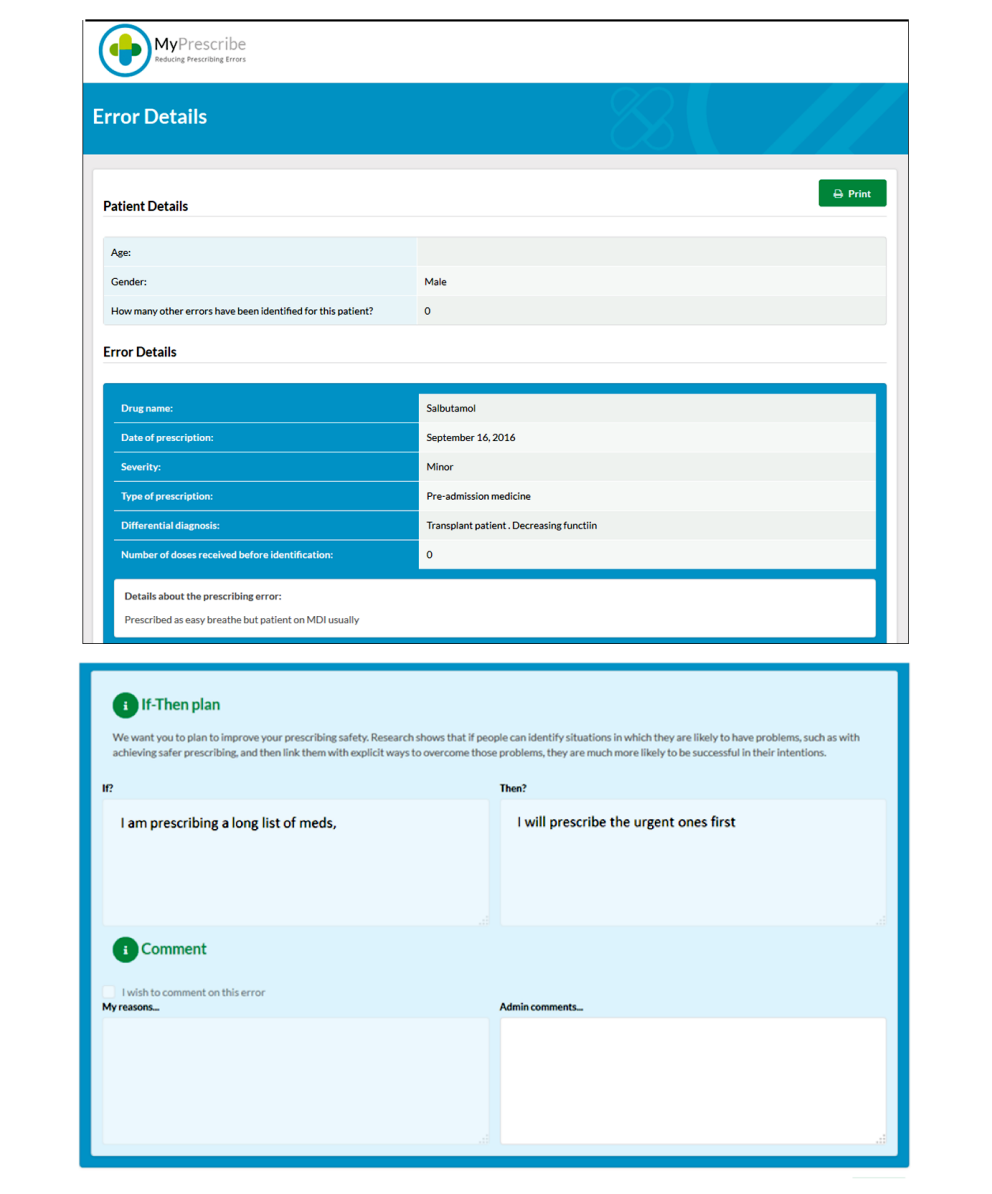
Screenshots of MyPrescribe user interface.

MyPrescribe is a mobile-compatible website that delivers feedback on prescribing errors in an appropriate manner to both medical and nonmedical prescribers and enables implementation intentions [17] (ie, what to do differently in future occasions) to be used without the need for debriefs with expert input. Throughout the development of MyPrescribe, a series of workshops with pharmacists and junior doctors were conducted to ensure that the most appropriate technological solution was developed for prescribers working in acute care trusts. Regular meetings were conducted with clinical pharmacists working on wards to ensure that data collection integrated with their existing workflow. Prescribing error data were collected by clinical pharmacists at the study sites using a previously developed data collection tool, Form^2^[34], for use on an Apple iPad. This allowed ease of data collection and transfer of information to MyPrescribe. A unique identifier was used to send the information from Form^2^to MyPrescribe. Doctors could log in and work through a series of screens, where they were presented with details of their prescribing error and asked to construct a personalized implementation intention as to how they planned to prevent such an error from occurring in the future. Relevant screenshots from MyPrescribe are presented in Figure 2.

### Platform and Browser Compatibility

Since October 2004, all websites must meet the World Wide Web Consortium (W3C) specification for accessibility to comply with the UK Government Disability Discrimination Act 1995. The website was developed to conform to the W3C standard of HTML5, where possible, as well as CSS 3.0. The website was developed to meet the W3C’s Web Accessibility Initiative level A specification and therefore was fully functional in browsers that comply with W3C standards, including Internet Explorer, Firefox, Chrome, Safari, and Edge. As a standard, the website designers HMA (Health Marketing Agency) checked browser compatibility on Internet Explorer 10+ as well as the last 2 versions of Firefox, Chrome, and Safari. The website was fully functional on previous versions of these browsers as well as those not listed. However, some styling may vary for these browser types.

The website was developed to conform to NHS software requirements and security systems. Data security during the transfer between the device and the server was achieved by using Transport Layer Security/Secure Sockets Layer for all communication. This is a cryptographic protocol that is designed to protect against eavesdropping, tampering, and message forgery, which is also used for Web-based banking transactions. Data are stored on the Amazon Web Service (AWS), which has the strictest and most evolved IT compliance standards globally. The website uses the AWS servers in Ireland, which comply with European regulations on data protection.

### Participants and Methods

Pharmacists and junior doctors (foundation year 1 [FY1] and foundation year 2 [FY2]) were recruited from two large NHS Foundation Trust hospitals in Greater Manchester. To obtain a sufficient amount of data, pharmacists (n=11) were invited to collect prescribing error data for junior doctors (n=52) over a 4-month period. The two trusts provided two different environments (electronic and paper-based prescribing) to maximize the potential for the website to be rolled out more broadly to other hospitals at a later stage.

A subsample of foundation doctors recruited though convenience sampling was asked to trial the website using data collected by clinical pharmacists with whom they usually worked. The participants were asked to log into the website, view a series of errors, and asked to interact with the website, thereby engaging with all the components. The same group of participants was then invited to take part in semistructured interviews exploring the perceptions of the acceptability and feasibility of MyPrescribe as a training tool aimed at improving prescribing practices. The interview was conducted immediately after the participants had used the intervention (within a 24-hour period) to aid recall of the specific errors identified and the specific perceptions of using the system. Participants were aware of both aspects of the study beforehand. The topic guide was developed to address each component of the COM-B model to gain insights into the key issues associated with the prescribing practices and the implementation of MyPrescribe. The topic guide explored three key areas, including (1) the extent to which this intervention could be integrated into daily practice, (2) the acceptability of how a psychological theory (implementation intentions) had been used to inform MyPrescribe, and (3) the perceptions of whether this intervention could reduce prescribing errors generally.

The potential participants were identified by the members of the pharmacy team at each study site and sent an invitation to be a part of the study. The doctors who were interested were provided with a participant information sheet outlining the purpose of the study, and their written consent was obtained. The recruitment strategy used a purposive sample to ensure maximum variation in terms of the grade of the doctor (FY1 and FY2), hospital site, and clinical specialty. The study received governance approvals from a local R&D approval office (ref 191058) and a university research ethics committee (ref 15541).

### Data Analysis: Mapping Findings to the COM-B System

Interviews were transcribed verbatim, and NVivo was used to code and categorize the data. Analysis was informed by the principles of framework analysis [35], with findings mapped to the components of the COM-B model. This approach was chosen, as it enabled both predetermined and emergent issues to be explored in depth while using the COM-B model as an explanatory framework. It is particularly useful for research in applied health service settings. Initial coding was carried out by one of the authors (CK) and themes were discussed and agreed upon with a second study author (MPT), whereas the emerging theoretical concepts and issues were agreed upon by all study authors. After an agreement was reached, the themes and code names were matched to the relevant domains of the COM-B model, which included capability, opportunity, and motivation. This involved rereading the data relating to each code and mapping them to the appropriate domain within the model.

To maximize trustworthiness of the data analysis, researcher triangulation was used, which employed a range of perspectives from within the research team to discuss and interpret the data [36]. The emerging themes were discussed with the team members, each from a different background, including pharmacy practice, health psychology, and health services research. This process reduced bias and ensured that the findings were verified and the appropriate interpretation given.

## Results

**Figure 3:**
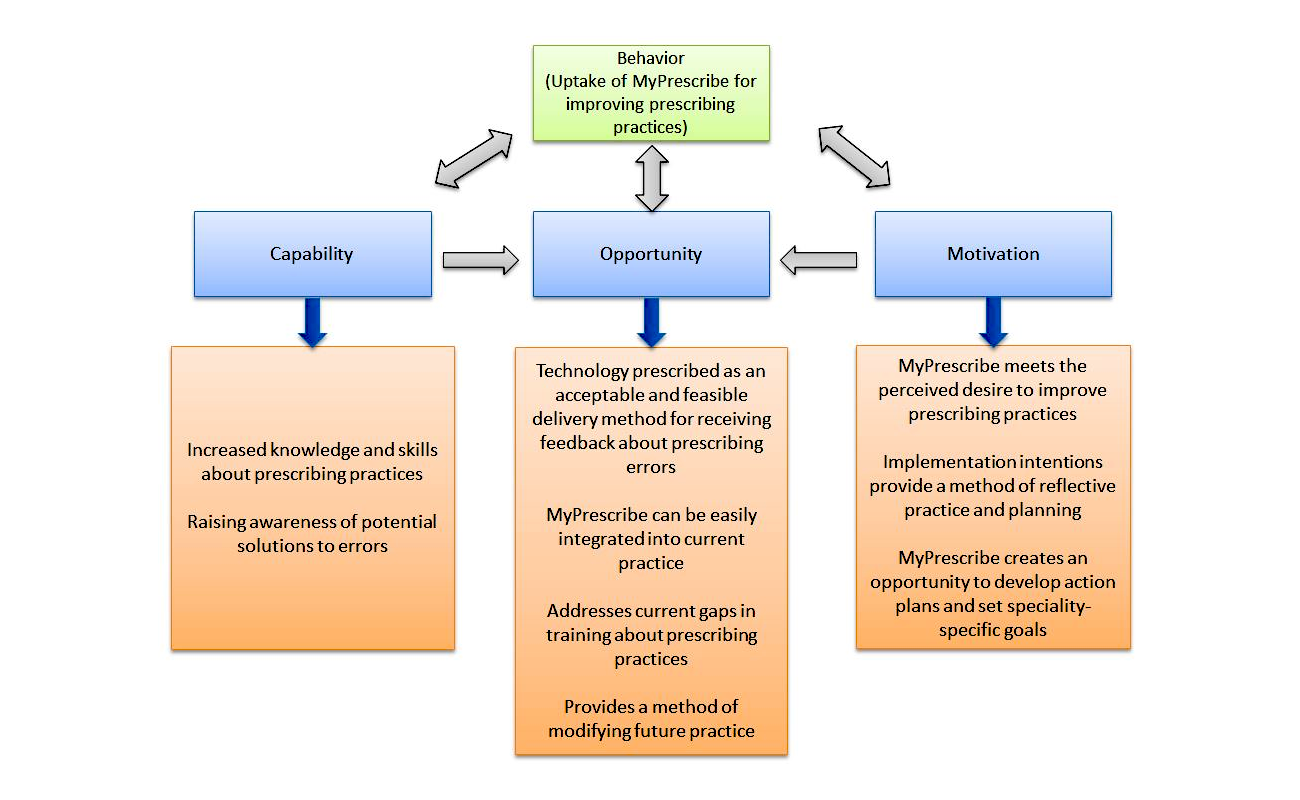
The acceptability and feasibility of MyPrescribe mapped to the components of the COM-B (capability, opportunity, motivation, and behavior) model.

Across the two hospitals, pharmacists (n=11) collected data relating to 200 prescribing errors for 52 FY1 and FY2 doctors (mean=3.9 errors per doctor; range=1-11 errors). A total of 15 FY1 and FY2 doctors (FY1: n=9; FY2: n=6) were recruited from the study sites to take part in a face-to-face semistructured interview (males: n=4; females: n=11). The participants were from a range of specialties/wards, including Heart Care (n=5), Renal transplant/renal (n=6), Gastroenterology (n=3), and mixed specialties (n=1). We limited our demographic information to maintain confidentiality. Interviews ranged from 20 min to 38 min, with a mean length of 27 min. The findings are presented according to the four major themes identified, which have been mapped onto the three components of the COM-B model [27], as illustrated in Figure 3. Illustrative quotes are presented verbatim, with unique participant IDs (allocated in order of the interview) presented alongside.

### Domain 1: Capability

#### Current Feedback Insufficient to Change Prescribing Practices

Doctors reported a desire to improve their prescribing practices as a part of their continued professional development. Developing their knowledge and skills relating to prescribing practice was perceived as important, both in terms of raising awareness of past mistakes and taking steps to improve future practice by keeping errors to a minimum. One of the participants notes:

I think it’s really important to be able to think about why you’re doing...’cause everybody makes some kind of error...mistake, at one point, but it’s if you think about it, then you can minimize the chances of it happening again.

P7; FY1

Barriers to improving their professional practice relating to prescribing behaviors were also highlighted. Participants reported that opportunities to develop more advanced prescribing skills were hampered by the inadequacies of current feedback on prescribing errors as a part of routine practice. Doctors were not always informed by the clinical pharmacist about the errors they had made. Errors were often corrected by a colleague on another shift with little or no explanation of the error. Consequently, there were limited opportunities to increase their knowledge and skills about appropriate prescribing practices. The participants noted:

A lot of the time with F1 [FY1] s, especially if you’re seeing people who you don’t see on a regular basis, you’d be writing Kardexes [in-patient prescription charts] or prescribing things like anti-emetics or sleeping tablets or whatever and actually you never see that patient again. So if you have made an error there’s no way you’re ever going to know unless someone tells you. I mean there’s definitely situations where I’ve probably made errors and don’t know about it and I’ve seen errors made by colleagues that will never know about it because they never went back to that patient.

P10; FY1

#### Attitudes Toward and Approaches to Prescribing

MyPrescribe was perceived as a useful tool for changing the ways doctors approach prescribing. This was both in terms of identifying unhelpful patterns in current practice, as well as enabling doctors to think in a more structured way about future practice. An important potential consequence of using MyPrescribe was equipping doctors with the knowledge and skills to identify the possible solutions to challenges they faced in prescribing practices through the application of “If-Then” plans to situations where an error had been made. One of the participants stated:

Yeah, I think if it comes up and especially if you tend to make certain errors more common than others, you can pick up on patterns and what you think, like what our common mistakes are, and then when I think about situation like oh yeah, I always do this when I try and be on the phone and do this at the same time. You tend to realize that the behavior that you might not pick up on.

P13; FY1

Participants were aware of the impact prescribing errors had on patient care. MyPrescribe was perceived as a way of increasing awareness of the implications of making errors and the importance of being informed of any errors made. Consequently, MyPrescribe was a way of changing attitudes toward prescribing practices. Another participant noted:

But personally, I think it would change my attitude to prescribing. I’d probably be a bit more wary on the things that I’ve made a mistake on before, and things like that. Because even now, I probably have made mistakes, and sometimes [the clinical pharmacist] just corrects them, or someone else corrects them without telling me, and I won’t know, and I’ll probably make that mistake in the future. So if I’d got this system, I’d know all the mistakes that I’ve…potentially. So that would be good.

P11; FY1

MyPrescribe was perceived as a way of ensuring safer prescribing/patient safety through a more transparent error feedback process. This also created more efficient working practices such as saving time for both the pharmacists and the doctors, as well identifying opportunities to minimize errors made by less experienced doctors:

It’ll make you think more and probably mean that I’d make less mistakes in the future because I’ll be thinking and it’s safer. It will save the pharmacist time, save me time, all the patients get treated faster I guess.

P10; FY1

So, it’s nice to have something like this where you can, hopefully, very quickly, get some data. Get some feedback about how you’ve been prescribing, and hopefully there’s nothing too serious, but certainly, things that will stop you from doing something that serious.

P2; FY1

### Domain 2: Opportunity

#### Technology Perceived as a Way of Delivering Timely and Effective Feedback to Health Professionals

Participants described how technology supported their practice generally, reporting how technology allowed them to recognize errors and reflect on past mistakes in their own time in a nonthreatening way. Timing was highlighted as an important issue, not only in terms of receiving timely feedback on their own practice but also at a critical point during their foundation year training period. The participants noted:

I’d probably do it from home once a week and set aside one evening when I was going to log in and do it, just so that then I know that I’m not going to be disturbed, I’ve got no-one looking over my shoulder and then I can do the work that I need to do related to, if I’ve made any errors and where they were made.

P1; FY2

I think mostly F1s [FY1s], F2s [FY2s] now, would like that. Especially in their first couple of years when you are getting used to like what’s right and patients I think.

P14; FY1

MyPrescribe was perceived as an important learning resource that strengthened junior doctors’ current e-learning strategies. This was seen as a way of complementing existing learning tools that focused on critical reflection and satisfying the requirements of their e-portfolio (a tool for recording career progression, professional development, and evidence illustration training competencies). One of the participants said:

Yeah, I think when people have to do their portfolio thing, they more likely look into this, because you can...I think also it’s very useful if this can connect to our e-portfolio somehow...it would be great, because then we could use it as evidence in certain situations that, you know, when you’re seeing this patient has errors and acted on it.

P13; FY1

More generally, technology was a feasible and acceptable delivery method for techniques to improve prescribing practices by modifying future behavior. MyPrescribe was perceived as a positive addition to a range of apps currently used by junior doctors, allowing it to be easily integrated into their routine practice. Consequently, participants reported that this would lead to improvements in working practices:

I think it’s easier than paper, especially if you’re busy and you just have it to hand, I think it’s rather nice and then you can access it, you know, anytime and you don’t have to be on the ward or...you know. Yeah I think the app in itself is a good idea. We use apps all the time already.

P7; FY1

### Domain 3: Motivation

#### Impact of Implementation Intentions

Participants suggested that MyPrescribe was a way of identifying areas of their clinical practice that could be improved, particularly in relation to specialty-specific prescribing. A key factor for successful implementation as reported by the participants was that the intervention addressed knowledge gaps in their training about prescribing practices. This allowed doctors to think more critically as well as consciously about their prescribing:

Well, if I’m making errors related to, I don’t know, a certain subset of medications related to a certain specialty, say I’d been finding it difficult with prescribing cardiac drugs, you know, it’s going to make you look further, not only into the pharmacology in that area but then the conditions you’re treating with those medicines. So actually it’s going to help you with a whole range of things.

P1; FY11

Implementation intentions were perceived as an effective method for encouraging more reflective practice. This was particularly important in the context of a busy clinical environment that maximizes the chances of errors being made and limits the time for critical reflection because of an increased workload and a high turnover of patients. One of the participants stated:

It would encourage me to reflect and think about it more when the pharmacist tells me, oh you’ve done this, nothing…just something minor. I’m like, okay I’ll change it and I couldn’t even tell you…I couldn’t tell you one now. Nothing sticks out in my mind that I’ve done minor because you fix it and you forget about it. So maybe logging it, anything conceived and repeated the same sort of things, and it probably would change.

P14; FY1

Participants described specific ways in which “If-Then” plans could be used for modifying future practice. This involved knowing how the identification of specific errors creates opportunities to learn from previous mistakes. Having a system of documenting previous mistakes in place, especially minor errors that were not routinely remembered, and possible solutions, was seen as particularly important for enabling more structured ways of reflecting on practice. The participants were able to provide examples of how “If-Then” plans could be used in specific areas of clinical practice. This was seen as a way of ensuring repeated mistakes were minimized and also as a prompt for future situations where prescribing is a challenge:

Participants described the reflective processes they were able to engage in as a result of using MyPrescribe. Action planning and goal setting were highlighted as two important decision-making processes they were able to engage in for prescribing behavior. This allowed them to think about their past prescribing behavior and practice more generally. It was perceived that MyPrescribe, and the implementation intentions in general, could integrate into (and complement) existing training. A participant noted:

So if I make a mistake…all right, if I’m in a situation where I could potentially make a mistake, these are the things I need to do to avoid those errors. I like it because it’s simple to fill in, but it’s also, you’re creating an action plan at the same time. So you’re reflecting and action planning at the same time. So, again, it’s about efficiency.

P2; FY1

### Domain 4: Behavior

#### Creating a More Structured, Reflective Approach to Health Professional Practice

Feedback about current practice was perceived as important for highlighting areas of junior doctors’ day-to-day practice that could be improved. Some participants were driven to change their own behavior by the desire to keep prescribing errors to a minimum:

Well, personally, I want to not make mistakes, which I think anything that improves your prescribing practice is only a good thing.

P9; FY1

I think people who have got portfolio things to do…prescribing’s a big thing in the new curriculum, for the foundation so, I think anything that can ensure that you’re thinking more about prescribing and changing what you’re doing is going to be popular.

P8; FY1

Participants described specific ways that MyPrescribe translated into behavior change in terms of changing specific prescribing behaviors. Doctors reported that implementation intentions provided a way of transforming critical reflection into practice change by highlighting solutions to a problem (action planning) and how this could be implemented in day-to-day practice (action):

It’s useful to think about a solution to the problem, so if this…if I’m in the situation then this is how I’m going to tackle it and then put it into action.

P1; FY2

So I’ve prescribed something then…it’s been wrong, maybe too high a dose or something. And then it’s been flagged up to me that it was wrong, and then obviously I’d go back to this and I’ll know not to do that in the future.

P5; FY2

One of the major perceived barriers to practice change was the heavy workload faced by junior doctors. This was particularly important for working in different specialties or settings that pose different challenges in terms of prescribing practices. MyPrescribe was perceived to facilitate professional behavior change by providing the platform to a more structured, reflective approach to prescribing:

I think it’s a good approach to take, especially for prescribing. It makes you think about the different situations that you’re prescribing in and the different external things that impact on your prescribing, which is easy to overlook when you’re busy..

P1; FY2

You are kind of enabled to think about your prescribing more, I think people should become more comfortable with prescribing the more they use it. If it’s helping improve their practice...I think it will probably help people to see prescribing as a much more structured activity and to think about it actively more from this.

P7; FY1

## Discussion

### Principal Findings

This paper describes the development of a novel theory-based technological innovation aimed at reducing prescribing errors by foundation doctors. To our knowledge, this is the first study to examine this type of intervention specifically for prescribing behaviors, using a recognized theoretical framework such as implementation intentions [16]. MyPrescribe was perceived as a highly acceptable and feasible delivery method of providing doctors with information about prescribing errors, as well as providing opportunities to construct personalized implementation intentions aiming at modifying future practice.

The COM-B model, which focused specifically on understanding the key elements of intervention design and explaining target behaviors [27,28] identified the barriers and enablers to the uptake of MyPrescribe and the specific mechanisms through which the intervention operates (see Figure 3). First, MyPrescribe was perceived as a way of increasing knowledge and skills about prescribing practices by identifying prescribing errors, and more importantly, raising awareness of potential solutions (*capability*). Second, technology was perceived as a feasible and acceptable vehicle for both delivering and receiving feedback about prescribing errors. This was seen as being critical in terms of ongoing professional development, addressing gaps in current training about prescribing practices, and modifying future clinical practice (*opportunity*). Third, implementation intentions provided a method of conscious, reflective planning, which was particularly important in the context of changing prescribing behaviors. Participants were able to think more critically about their practice and create action plans to modify future practice. Consequently, participants were motivated to improve their prescribing practices (*motivation*).

Although it is primarily the foundation doctors who undertake the majority of prescribing in hospitals, they are rarely given feedback on their prescribing errors [12]. Current feedback methods for prescribing range from formal audit and feedback interventions [7] to the more informal routine feedback as part of day-to-day clinical practice [12], or “ad hoc” feedback as errors are identified [5,12]. Doctors often use pharmacists as a prescribing “safety net” [5,16,37], which consequently limits opportunities for professional development and can cause avoidable stress in the early stages of clinical practice [38]. Common features of previous feedback interventions include limited opportunities for personal reflection about one’s mistakes and the platform to create personal action plans for professional development. MyPrescribe demonstrates a feasible and acceptable way of delivering feedback on prescribing errors aimed at improving future practice by addressing these known barriers. The participants in this study expressed concerns about gaps in prescribing teaching as has been seen elsewhere [5], which MyPrescribe was perceived to address. Additionally, the challenges in evaluating eHealth applications, particularly around engagement with interventions, have been well documented [39]. MyPrescribe was perceived to overcome such barriers because the participants reported that the intervention was a way of complementing current training tools. Technology-specific barriers to using Web-based interventions to facilitate professional practice such as time and organizational constraints [40] were also perceived to be addressed.

By including implementation intentions as a specific evidence-based theoretical framework [41], we have provided recommendations to inform the design and delivery of future interventions that would help improve prescribing practices. Implementation intentions have been widely used for a range of patient/public behavior change strategies with a high degree of success [19]. Our study demonstrates that this strategy is acceptable and feasible in the context of prescribing practices as a part of health professional behavior change, a growing area in the context of evidence-based behavior change interventions. Our findings suggested that the participants were able to develop specific skills that could be mapped to an existing framework of behavior change techniques (BCTs), which included goal setting (BCT 1.1) and action planning (BCT 1.4) [27,28]. The precise mechanisms through which implementation intentions work in the context of health professional behavior change have been suggested, which helps to explain how this can be applied to prescribing practices. When forming action plans, health professionals are able to create a conscious mental link between a contextual cue (ie, a prescribing situation) and goal-directed behaviors (ie, appropriate prescribing). Health professionals may be more likely to perform the behavior as an automatic response [42,43]. Using the COM-B model has identified the behavioral determinants of prescribing behavior change and implementation of MyPrescribe. Interventions that aim to target prescribing practices must build on this work by clearly specifying intervention functions most relevant to this area of clinical practice.

### Strengths and Limitations

This is the first study to develop a theory-based technological intervention aimed at improving the prescribing practices of foundation doctors. The involvement of key health professionals (pharmacists and foundation doctors) at all stages of the development process ensured the creation of an intervention that could easily be integrated into their busy day-to-day practice. By including implementation intentions as the key theoretical framework for the intervention and explaining the perceived mechanisms behind the intervention using the COM-B model, this allowed for a more detailed understanding of how the intervention works in practice, thereby satisfying the first phase of developing interventions according to a recognized and widely used framework [44].

However, there are limitations that must be considered in light of our findings. The intervention has not yet been tested to investigate whether the perceived impact translates into actual impact on prescribing errors. This study is at the development stage of evaluating complex interventions, where the Medical Research Council guidance has suggested that it is essential to initially “develop the intervention to the point where it can reasonably be expected to have a worthwhile effect” [44]. Qualitative investigations have teased out the ways in which the intervention *could* work. Future research will continue the evaluation process, with feasibility studies leading to evaluation studies of effectiveness and cost-effectiveness, a necessary component needed to draw firm conclusions about the effect of MyPrescribe on reducing prescribing errors.

### Conclusions

This paper described the development of MyPrescribe, a novel technological intervention aimed at improving the prescribing practices of foundation doctors. In summary, implementation intentions provide the theoretical foundations on which information about prescribing errors should be delivered and present opportunities for prescribers to formulate solutions to past and future errors. MyPrescribe could make a valuable addition to medical prescribers’ training in reflective practice.

## Abbreviations

AWS: Amazon Web Service
BCTs: behavior change techniques
COM-B: capability, opportunity, motivation, and behavior
FY: foundation year
NHS: National Health Service
W3C: World Wide Web Consortium
